# The Input of Structural Vaccinology in the Search for Vaccines against Bunyaviruses

**DOI:** 10.3390/v13091766

**Published:** 2021-09-04

**Authors:** Alexandra Serris

**Affiliations:** 1 Department of Infectious Diseases and Tropical Medicine, Hôpital Universitaire Necker-Enfants Malades, Assistance Publique—Hôpitaux de Paris, Université de Paris, 75015 Paris, France; alexandra.serris@aphp.fr; 2 Structural Virology Unit, Institut Pasteur, 75015 Paris, France

**Keywords:** bunyavirus, structural vaccinology, hantavirus, phlebovirus, nairovirus, neutralizing antibodies, structure-based vaccine design

## Abstract

A significant increase in the number of viruses causing unexpected illnesses and epidemics among humans, wildlife and livestock has been observed in recent years. These new or re-emerging viruses have often caught the scientific community off-guard, without sufficient knowledge to combat them, as shown by the current coronavirus pandemic. The bunyaviruses, together with the flaviviruses and filoviruses, are the major etiological agents of viral hemorrhagic fever, and several of them have been listed as priority pathogens by the World Health Organization for which insufficient countermeasures exist. Based on new techniques allowing rapid analysis of the repertoire of protective antibodies induced during infection, combined with atomic-level structural information on viral surface proteins, structural vaccinology is now instrumental in the combat against newly emerging threats, as it allows rapid rational design of novel vaccine antigens. Here, we discuss the contribution of structural vaccinology and the current challenges that remain in the search for an efficient vaccine against some of the deadliest bunyaviruses.

## 1. The Concept of Structural Vaccinology

A fundamental aspect in the development of efficient vaccines is to understand what antigens will elicit an optimal immune response. In recent years, progress in the fields of human immunology and structural biology facilitated the generation of structural data on protein complexes and led to the development of a new approach to design better immunogens, commonly referred to as *structural vaccinology*. All vaccines in routine use, except BCG (which is believed to induce T cell responses), are thought to mainly confer protection through the induction of antibodies, in particular neutralizing antibodies (nAb), i.e., antibodies able to prevent the infection of a target cell. With the development of single B-cell sequencing, neutralizing monoclonal antibodies (mAbs) can be isolated from convalescent individuals and their targets identified. Structural vaccinology is a rational process that involves three steps: elucidating atomic-level structures of viral antigens either alone or through interactions with neutralizing antibodies (with X-ray crystallography or electron microscopy), the use of this structural information to engineer modified antigens stabilized in an immunologically relevant conformation and the incorporation of these re-engineered antigens into one of the vaccine platforms (such as subunit, viral vector or DNA/RNA vaccine platforms) to produce vaccines with enhanced immunogenicity and breadth of coverage [1]. Indeed, analysis of the interactions between neutralizing mAbs and viral antigens allows for: (1) precise mapping of crucial epitopes, (2) inference of the sequence modifications needed to stabilize a desirable conformation or to modify the surface in order to display the preferred epitopes and (3) identification of structural similarities among epitopes found within a given class of viruses, which can be used to build a vaccine active against all members of this class.

In the case of enveloped viruses, such as bunyaviruses, neutralizing antibodies usually target the viral envelope proteins that are responsible for receptor recognition and/or inducing fusion of the viral and cellular membranes [2]. These glycoproteins, in particular the membrane fusion proteins, are difficult to study because they are present in a labile form at the surface of the virions. Indeed, fusion proteins adopt two conformations: a metastable pre-fusion conformation that mediates viral entry, and a stable post-fusion conformation that is formed after membrane fusion has occurred. It is the irreversible conformational change from pre- to post-fusion conformation that provides the energy required to overcome the high kinetic barrier of membrane fusion. Neutralizing antibodies sometimes recognize both conformations, but those specific to the pre-fusion conformation are far more potent [3]. As exemplified by the history of failed Respiratory Syncytial Virus (RSV) vaccines, the harsh inactivation protocols used to produced safe, inactivated vaccines often result in vaccine candidates that contain mostly the post-fusion conformation, incapable of eliciting protective neutralizing Abs [4]. The same phenomenon is observed with other vaccine development platforms that contain/express the native envelope proteins (e.g., subunit, viral vector or DNA/RNA vaccines). Indeed, as the prefusion conformation is metastable, it needs to be stabilized to be incorporated into a candidate vaccine. The high-resolution structures of several fusion proteins (such as RSV or more recently SARS CoV-2 spike protein), either alone or in complexes with neutralizing mAbs, have allowed scientists to rationally identify sites (e.g., hydrophobic cavities, etc.) where mutations might be introduced in order to stabilize the most immunologically relevant conformation of these proteins [5]. The RSV fusion protein F was stabilized in its pre-fusion conformation by adding cysteine residues and filling hydrophobic cavities (a group of mutations termed DS-Cav1) [6]. This DS-Cav1 stabilized F protein showed promising results when used in a sub-unit vaccine candidate [7,8]. The sequence of a prefusion-stabilized SARS-CoV-2 spike protein is encoded in the licensed RNA-based vaccines mRNA-1273 and BNT162b [9]. Compared to RSV and SARS-CoV1, whose envelope glycoproteins assemble as independent spikes allowing the study of their isolated entities, bunyaviruses present a further challenge as their envelope proteins form complex lattices covering the viral surface. To decipher the molecular determinants of humoral protection, the interaction of antibodies with the whole assembly is important to understand.

## 2. The Surface Glycoprotein Lattice of Bunyavirus Particles Constitutes a Challenge for Vaccine Design

The order Bunyavirales constitutes a large group of enveloped RNA viruses distributed worldwide and classified into 12 families [10]. This review will focus on the input of structural vaccinology in the search for a vaccine against four bunyavirus families that pose a significant threat to human health: *Hantaviridae*, *Phenuiviridae*, *Nairoviridae*, and *Peribunyaviridae*. In 2018, the *Arenaviridae* family was reclassified in the bunyavirales order. As Arenavirus surface glycoproteins are not related to those of other bunyaviruses, they will be discussed in a separated part of the review.

**Figure 1:**
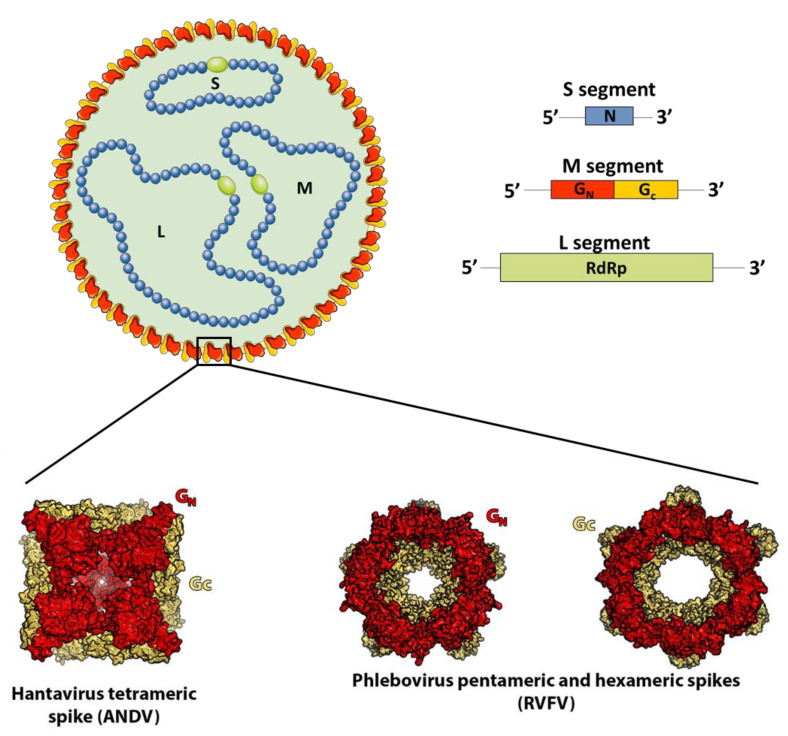
Schematic representation of a bunyavirus virion and main features of bunyavirus genomes (*Arenaviridae* family excluded). The genomes of most families belonging to the *Bunyavirales* order are divided in three segments. Based on the length of their coding sequences, these genomic segments are named large (L), medium (M), and small (S). The L segment encodes the RNA-dependent RNA polymerase (RdRp protein), the S segment the nucleocapsid protein (N) that covers the genomic RNA, and the M segment codes for a glycoprotein precursor that is post-translationally cleaved into at least two structural proteins, termed Gn and Gc. In addition, depending on the bunyaviral family, the genomic segments encode additional proteins, in general non-structural, which are not represented here. The structures of hantavirus (PDB: 6ZJM) and phlebovirus (PDB: 6F9F and 6F9C) spikes obtained via X-ray crystallography are shown in the lower panel. Gn is in red, Gc in yellow. The atomic-level structure of nairovirus and orthobunyavirus spikes is not yet understood.

The abovementioned bunyaviruses are enveloped viruses with a genome formed by three segments of single-stranded RNA of negative or ambi-sense polarity. The virions are coated with two glycoproteins termed Gn and Gc (Figure 1).

**Figure 2:**
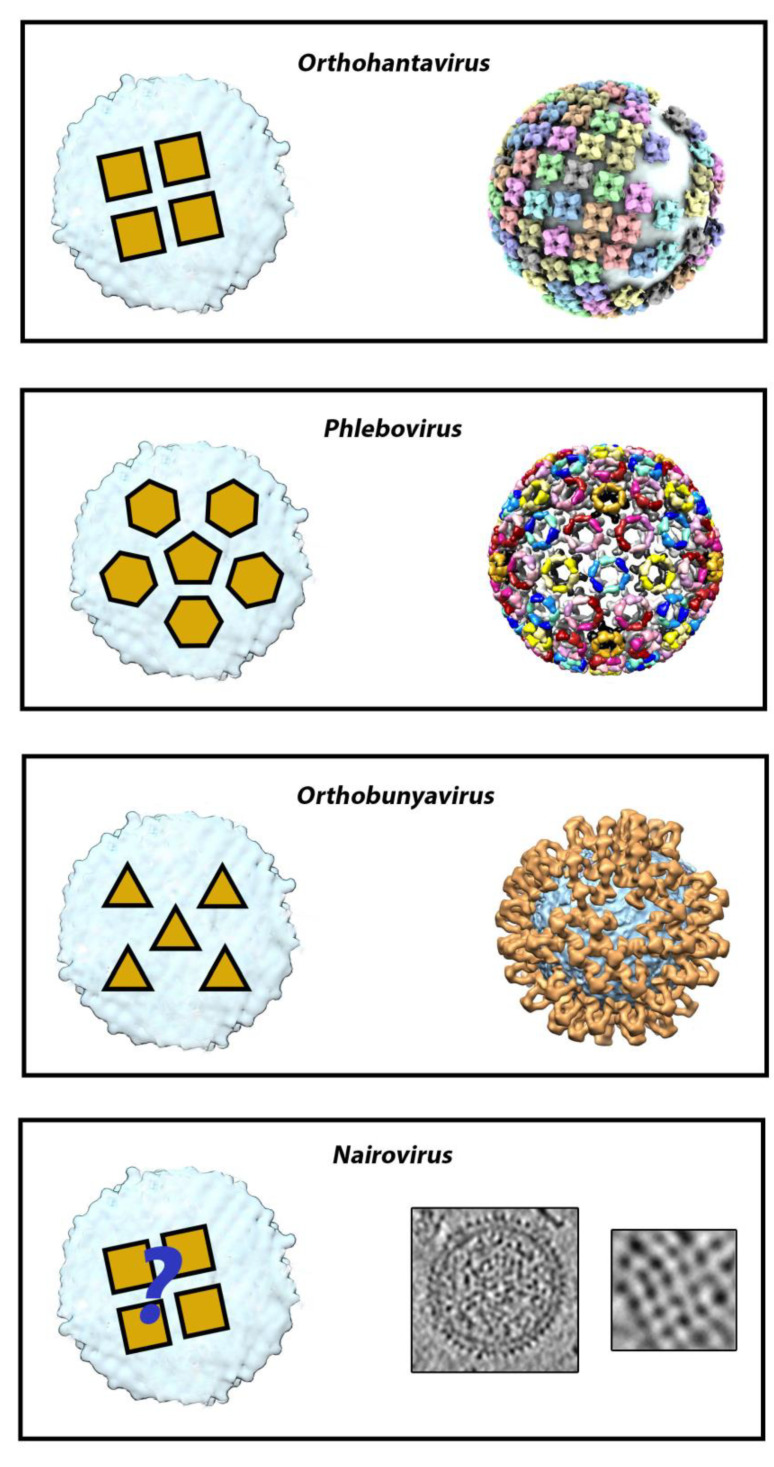
Architectures of the outer lattice and spike organization of orthohantavirus (Hantaviridae), phlebovirus (Phenuiviridae), orthobunyavirus (Peribunyaviridae) and nairovirus (Nairoviridae) (adapted from [12]). Orthohantavirus virions are pleomorphic and covered by a grid-like pattern formed by tetrameric spikes of Gn/Gc [13,14]. Reconstruction of a hantavirus particle obtained by fitting an Andes virus glycoprotein spike into a Tula virus reconstruction (PDB: 6ZJM). Each (Gn/Gc)_4_ spike is displayed in a different color. Virions of phleboviruses display a T = 12 icosahedral lattice formed by 110 hexameric and 12 pentameric capsomers consisting in equimolar amounts of Gn and Gc [15]. Model of a Rift Valley fever virus particle (PDB 6f9b) from [2]. The surface of peribunyaviruses is decorated with tripod-like projections formed by Gn/Gc protomers [16]. Tomographic reconstruction of a Bunyamwera virus particle from [12]. The structure of nairovirus virions has not been elucidated so far. They present a pleomorphic morphology similar to hantaviruses from the cryoEM study [17]. The cryoEM images are from [12].

Gc is the fusion protein (belonging to the class II fusion proteins), and Gn is a “accompanying” protein required to control Gc proper folding in the secretory pathway and prevent its premature activation [11]. Both glycoproteins interact co-translationally to form a metastable spike comprising three or four Gn/Gc heterodimers, depending on the family. During viral budding, the spikes interact with one another to shape an outer lattice covering the virus, which plays a major role in particle assembly, mediates all of the entry steps into permissive cells, and is the sole target of neutralizing antibodies [11]. In spite of structural and sequence analyses suggesting that both Gn and Gc share a similar fold and a common ancestor [11], the architecture of the outer lattice is a distinctive feature of each family (Figure 1 and Figure 2).

As the outer lattices have different organizations, different parts of Gn and Gc are exposed at the virion surface and the target of the humoral response varies according to each bunyavirus family. So far, isolated neutralizing antibodies against phlebovirus have been shown to target exclusively Gn [18], and those against nairoviruses and orthobunyaviruses target Gc [19,20] whereas neutralizing antibodies generated in the course of hantavirus infections bind either Gn, Gc or both [21,22]. As classical methods failed to produce efficient vaccines against bunyaviruses (such as live attenuated or inactivated vaccines), partially for the same reasons that have hampered RSV vaccine development, efforts have been made to obtain a better characterization of the molecular determinants of humoral protection using the structure of the viral glycoproteins alone and in complex with neutralizing antibodies.

## 3. *Orthohantaviruses* (*Hantaviridae* Family): An Example of the Importance of the Quaternary Spike Structure and the Global Lattice Organization

Hantaviruses are worldwide-distributed rodent-borne viruses [23]. They are categorized into two groups based on their pathogenesis and geographic distribution: in America, New World hantaviruses (such as Sin Nombre virus, Andes virus or New York-1 virus) cause a disease termed hantavirus pulmonary syndrome (HPS) whereas in Eurasia, Old World hantaviruses (such as Hantaan virus, Seoul virus or Dobrava-Belgrade virus) cause a different syndrome called hemorrhagic fever with renal syndrome (HFRS). Mortality exceeds 30% for HPS and ranges from <1% to 15% for HFRS [23]. Each hantavirus is carried by a specific rodent family, which explains their striking geographic distribution [23]. Cross-species transmission to humans usually results from inhalation of aerosolized excreta from chronically infected rodents. However, studies of several outbreaks in Argentina have revealed that at least one New World virus, Andes virus (ANDV), can be transmitted from person to person through inhalation of aerosolized virions [24] and from mother to child through breast milk [25]. Two inactivated hantavirus vaccines have been licensed for human use in China and Korea targeting two Old World hantaviruses (Hantaan and Seoul viruses) but failed to induce a long-lasting response in phase III and IV clinical trials [26]. As there is no FDA or EMA approved vaccine nor specific therapeutics available, hantaviruses have been identified as potential bioterrorism agents by the Centers for Disease Control and Prevention [27].

Several studies have confirmed the major role of humoral immunity in hantavirus infection protection. A high level of specific antibodies is associated with a better outcome in patients [28], and passive transfer of mAbs protected against a lethal challenge in a hamster model of Andes virus infection [29]. As there are over 28 different hantavirus strains implicated in human pathology, an ideal hantavirus vaccine should be able to protect against the most pathogenic ones, if not all. Currently, the most promising candidate vaccines are based on multivalent DNA vaccines containing the M segment of one to four hantaviruses (two of them are in phase I/II clinical trials) [30]. However, although serologic studies in humans and immunization trials in animals using these multivalent DNA vaccines may have confirmed a certain degree of cross-neutralization across the hantavirus species, this cross-neutralizing activity appears to display high variability, notably depending on the viruses used in the candidate vaccine [31,32].

**Figure 3:**
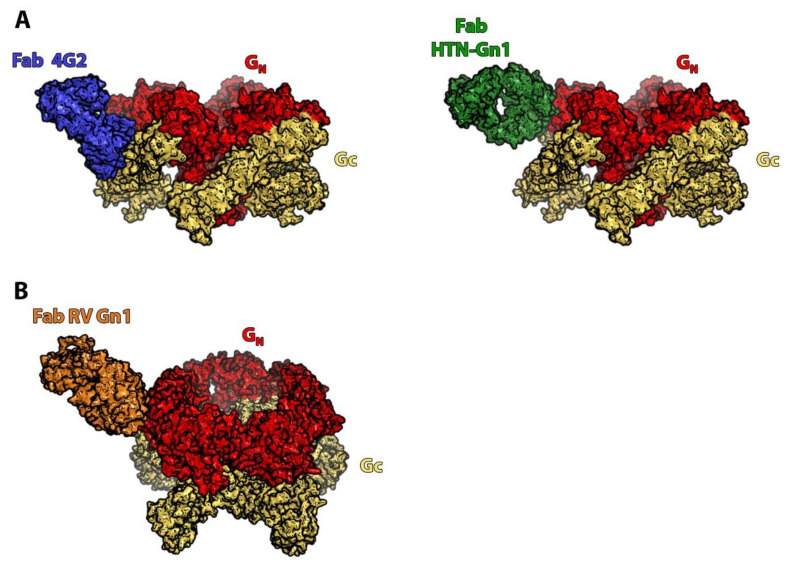
Orthohantavirus and phlebovirus spikes in complex with neutralizing mAbs. (**A**) Modelling of the interaction of Andes virus Gn/Gc spike with two different neutralizing antibodies. Gn is in red, Gc in yellow, Fab 4G2 in blue and Fab HTN-Gn1 in green. Both mAbs were obtained following experimental immunization of an animal model. mAb 4G2 targets Gc whereas mAb HTN-Gn1 recognizes Gn [34,36] (PDB: 6Z06 and 7NKS, respectively). (**B**) Modelling of the interaction of a RVFV pentameric spike with a neutralizing antibody. Gn is in red, Gc in yellow and Fab RV Gn1 in orange. RV Gn1 was also obtained following experimental immunization of an animal model. It targets Gn [37] (PDB: 6I9I).

Engdahl and colleagues began to decipher the molecular determinants of this variability, providing hints for how to overcome it. Using a panel of 20 mAbs from three donors previously infected with Sin Nombre virus (SNV) and 16 mAbs from one donor previously infected with ANDV, they showed that the variation in the breadth of the humoral response was linked to the different viral glycoproteins targeted by the mAbs: most of the broadly-reactive mAbs recognized Gc whereas the virus-specific mAbs targeted Gn [31]. This correlation was also observed in murine mAbs derived from B cells of immunized mice [22]. When mice were vaccinated with the glycoproteins of one virus species (in this study, ANDV) they produced a majority of Gn-reactive mAbs, whereas a sequential vaccination of the mice with three different hantavirus glycoproteins (from ANDV, Hantaan, and Puumala viruses) elicited the production of cross-reactive mAbs mainly targeting Gc, further suggesting a role of Gc-recognizing mAbs in a broadly reactive humoral response. These observations are in line with the difference in sequence conservation of each glycoprotein. Indeed, the Gc sequence is more conserved than that of Gn across hantaviruses [14]. This can be explained by both the glycoproteins’ function and their overall organization at the viral surface (Figure 3A). As mentioned, the hantaviral surface is coated by patches of tetrameric (Gn/Gc)_4_ spikes that form local lattices. Located at the top of the spike, Gn covers Gc and accounts for most of the solvent exposed surface of the virion; its sequence variability thus probably reflects the selection pressure by the host humoral immune response.

If the Gc sequence conservation and its major role in virus entry seem to indicate it as an ideal vaccine target, attention should be drawn to the fact that, in Engdahl et al.’s study, most Gc-specific mAbs were not found to be potent neutralizers of live viruses. Among the several explanations for this result, accessibility of the epitope to the immune system could be a major one. The accessibility of an epitope is of paramount importance in the design of a candidate vaccine, as it influences the antibody level of occupancy, i.e., the proportion of epitopes bound to their antibodies at the virion surface. If the epitope is poorly accessible, the level of occupancy will be low, a characteristic that has been associated with incomplete neutralization efficacy [33]. The two factors that can limit the accessibility of Gc epitopes are the location of Gc within the viral lattice and the lattice’s dynamic properties. First, the very position of Gc, close to the viral membrane, covered by Gn and interacting with neighboring spikes to its side, might limit the accessibility of its epitopes to the immune system. This is well illustrated by the recently described co-crystal structure of the mAb 4G2 in complex with Puumala Gc (Figure 3B) [34]. At the viral surface, the epitope of this cross-neutralizing mAb is covered by the neighboring spikes, and therefore only accessible on of some of the Gc molecules, on isolated spikes at lattice breaks. The second mentioned factor is the existence of a dynamic behavior of the spikes, called “breathing”. Indeed, it was recently shown that, at physiological temperature, independently of the target cell entry process, the spikes alternate between two conformations termed “open” (where Gn and Gc partially dissociate) and “closed” at the virion surface [35]. Importantly, only the “closed” form is functional, meaning that it can induce membrane fusion upon lowering of the pH. Although the relevance of this phenomenon has not been established in hantavirus infections, this dynamic feature of the spike may explain the incomplete neutralization activity of some Gc-targeting mAbs either by masking neutralizing epitopes or, alternatively, by transiently exposing decoy epitopes on Gc that are the target of weakly neutralizing mAbs.

Interestingly, Engdahl et al. isolated two mAbs which were potent neutralizers of both New and Old World viruses [31]. Binding experiments to either Gn- or Gc-soluble proteins revealed that none of them recognized Gn nor Gc alone, suggesting that the epitopes recognized by these two mAbs are formed or accessible only in the quaternary spike structure. Therefore, the best option to obtain an efficient pan-hantavirus vaccine would be to design a recombinant Gn/Gc spike, displaying the same quaternary antigenic sites as the native spike at the viral surface. An engineered spike, “locked” by structure-based mutagenesis to prevent any “breathing”, would probably be highly effective in eliciting a cross-neutralizing antibody response (Table 1). One should, however, be cautious when extrapolating data from static structures of crystallized proteins to model dynamic features of the spike at the surface of the viral particle. Indeed, in the native virion, the local constrains of the spike might modify the conformation or accessibility of the epitopes.

## 4. *Phleboviruses* (Family *Phenuiviridae*): Identifying the Precise Target of Neutralizing mAbs to Pave the Way for the Design of a Pan-Phlebovirus Vaccine

Phleboviruses belong to a genus of arthropod-borne viruses transmitted by sandflies, ticks and mosquitoes. The best-known representative is Rift Valley fever virus (RVFV), which primarily infects livestock, causing high rates of neonatal mortality and abortion, whereas human infection results in a wide variety of clinical outcomes, ranging from self-limiting febrile illness to ocular disease, meningoencephalitis or hemorrhagic fever [38]. RVFV distribution was initially restricted to the African continent but in recent years the virus has spread to Saudi Arabia, Mayotte, and Yemen [39]. The wide distribution of competent vectors in non-endemic areas and the potential impact of global climate change raise concerns about possible viral spread to Asia and Europe. As RVFV primarily affects domestic ruminants, the majority of human infections result from contact with blood or tissues of infected animals, and more rarely from mosquito bites or ingestion of contaminated raw milk [38]. Other human pathogens in the Phenuiviridae family include the Severe Fever with Thrombocytopenia virus, mainly described in China, and the sandfly fever Naples virus group (including notably the Toscana virus) that causes febrile illness and occasionally encephalitis in countries of the Mediterranean basin [40].

As the most efficient measure to contain RVFV spread is to vaccinate the animal source of the virus, research has focused mainly on veterinary vaccines. Whole inactivated virus or live-attenuated virus vaccines have been developed, but currently none of these candidates has been approved for human use. For humans, there are two vaccines currently defined as Investigational New Drugs in the USA: TSI-GSD-200 and MP-12 [39]. TSI GSD 200 is a formalin inactivated vaccine developed by the U.S. Army to protect at-risk laboratory workers, but it required several injections and did not elicit long-lasting immunity. MP-12 is a live-attenuated vaccine that has been conditionally licensed for animal use, although it has been associated with abortion in pregnant animals. Current research focuses on recombinant glycoprotein-based vaccines, DNA vaccines expressing the RVFV M segment, and replication-deficient virus vectors (all reviewed in detail in [41]). The use of a non-replicative vaccine against RVFV might be of particular interest as some authors have reported the detection of a re-assorted RVFV in a patient in South Africa potentially exposed to co-infection with live animal vaccine and wild virus [42]. The possibility of reassortment (i.e., exchange of segment) between vaccine virus and wild virus has important implications for the safety of the vaccine and its possible role in the evolution of RVFV. A better understanding the molecular determinants of humoral protection will likely be needed to improve the candidate vaccines, and some useful data can be drawn from the structural vaccinology field.

Indeed, so far, all RVFV neutralizing mAbs isolated from humans or generated in animal models target Gn (Figure 3) [18,43]. Moreover, in a study on mice vaccinated with cDNA expressing Gn or Gc, only Gn-vaccinated mice developed neutralizing antibodies, while Gc-vaccinated mice did not [44]. This immunodominant behavior can be explained by the exposed location of Gn at the top of the spike, covering Gc. Binding of neutralizing antibodies is thought to prevent infection either by blocking the attachment of virions to host cells [45] or by precluding RVFV glycoproteins of the Gn-Gc lattice and thus preventing exposure of viral fusion loops [37]. The fact that Gn is the main target of neutralizing antibodies might indicate that a vaccine containing Gn alone (such as a virus vectored or a DNA/RNA vaccine) could be sufficient to induce protection. Indeed, mAbs derived from B cells of rabbits immunized with Gn were protective in a mouse model [37].

Although amino acid differences in Gn/Gc proteins among RVFV strains can reach 2%, few studies have evaluated the cross-neutralization of different RVFV strains via antisera from vaccinated animals and it seems to vary according to the vaccine used [46]. Because of its exposed location at the top of the spike, Gn is under significant immune pressure, which can explain its sequence variability. Further work with different neutralizing antibodies will be necessary to map the distribution pattern of neutralizing epitopes on RVFV Gn and on other pathogenic phleboviruses. Indeed, despite the genetic and structural differences of Gn of RVFV and SFTSV, the isolation of two neutralizing mAbs targeting a similarly localized site on RVFV and SFTSV Gn, respectively, supports a common mechanism of neutralization and suggest the possibility to design a recombinant Gn that would induce immunity against several phleboviruses (Table 1). Indeed, as exemplified by Scarselli et al. with the factor H-binding protein (fHBP) of meningococcus B [47], the structure-based design of multiple immunodominant antigenic surfaces on a single protein scaffold represents an effective way to create broadly protective vaccines.

## 5. *Nairoviruses* (Family *Nairoviridae*): Protection Is Not Always Mediated Only by Neutralization

The main human pathogen in the nairoviridae family is the Crimean-Congo hemorrhagic fever virus (CCHFV), which is the most widespread tick-borne virus on earth. Hyalomma ticks are both the vector and the reservoir of CCHFV. In line with the wide geographic distribution of permissive ticks, cases of CCHF are reported throughout Eastern Europe, Africa, Middle East and parts of Asia. The geographic distribution of CCHF continues to increase, probably due to climate change: in 2016, Spain reported the first locally acquired human cases of CCHF, six years after the first evidence of virus circulation in the same area [48]. Infections occur through tick bite or contact with animals’ or patients’ infected body fluids. Mortality from CCHF can be as high as 30%. Based on phylogenetic comparison of their M segments, circulating strains of CCHFV have been classified into seven distinct clades. This genetic diversity represents a major challenge to the development of a broadly efficient CCHFV vaccine. Like RVFV, CCHFV has been included on the World Health Organization Blueprint list of viruses likely to cause major epidemics and for which no or insufficient countermeasures exist (https://www.who.int/activities/prioritizing-diseases-for-research-and-development-in-emergency-contexts (accessed on 22 August 2021).

There are currently no licensed vaccines against CCHFV. In Eastern Europe, a vaccine based on inactivated CCHFV has been used in endemic areas for military personnel, but its efficacy and safety profile are not optimal [49]. Several other vaccine candidates are currently being developed, in particular a DNA-vaccine containing the CCHV M segment [50,51]. The CCHFV M segment displays certain differences with that of phlebo- or hantaviruses. In addition to Gn and Gc, the nairovirus M segment encodes two other proteins, GP160/85 and GP38, whose functions are not clear yet [52]. Until recently, mAbs against CCHFV had only been isolated through experimental vaccination of mice [19]. These first studies had identified a particular pattern of recognition and protection among mAbs directed against CCHFV. Indeed, although only mAbs targeting Gc neutralized viral infection in vitro (and even cross-neutralized multiple strains), non-neutralizing mAb, targeting GP38, could confer protection against a CCHFV challenge in mouse models [53]. A recent study on a DNA candidate vaccine confirmed the significant role of GP38 in the vaccine immunogenicity and protection from a homologous CCHFV challenge in a mouse model [54]. Little is known about the function of GP38. It is a secreted protein, but it has been localized to the viral envelope and cellular plasma membranes of infected cells. This example highlights the fact that although neutralization is a very effective predictor of protection for many viruses, it is not the only one. The antibody effector functions mediated by the constant region of the heavy chain (Fc) are also potent mechanisms of protection; indeed, complete protection of mice through the mAb targeting GP38 required a functional complement activity [53]. An interesting parallel can be made with the nonstructural protein 1 (NS1) of flaviviruses. NS1 is a secreted viral protein whose blood level correlates with the severity of disease in Dengue infected patients [55]. NS1 has been localized on the membrane of target cells and can modulate the immune response by activating Toll-like receptor 4, disrupting endothelial barrier function and manipulating complement. Immunization with NS1 has been shown to elicit a protective humoral response against Yellow Fever, Dengue, and Tick-borne Encephalitis flaviviruses [56]. However, as GP38 sequences are more divergent among different CCHFV clades than those of the Gn/Gc glycoprotein complex, it is unclear if GP38-targeting can form the sole basis of broadly protective CCHF therapeutics.

A recent study on the human memory B cell response to natural infection in CCHF survivors from Uganda partially confirmed the results obtained with mice models. Among the 361 mAbs isolated, Fels et al. identified high-affinity binders to Gc or GP38 but none to Gn [57]. These results might reflect the topological arrangement of Gn and Gc on the viral particle. The nairovirus Gn/Gc spike structure has not been elucidated so far, but this apparent immunodominance of Gc over Gn suggests a similarity with its orthobunyavirus counterpart in quaternary organization (see below) with a higher surface exposure of Gc over Gn.

Fels et al. further mapped the different Gc epitopes by mutagenesis and found that 50% of the mAbs isolated from each donor targeted the same domain. Synergistic combinations of neutralizing mAbs targeting different domains of Gc were identified, suggesting a cooperative binding effect where the interaction of one antibody with Gc would modify Gc conformation and favor the binding of a second one. Indeed, enhancements in neutralization breadth and potency were attained by physically linking variable domains of synergistic neutralizing mAb pairs through bispecific antibody (bsAb) engineering. Furthermore, although several Gc-targeting neutralizing mAbs and non-neutralizing GP38-specific mAbs protected mice from lethal CCHFV challenge in pre- or post-exposure prophylactic settings, only a single bsAb afforded therapeutic protection. Structural characterization of Gc in complex with such a bsAb would provide very useful information for vaccine design: it could provide the molecular basis for the engineering of a recombinant Gc stabilized in its pre-fusion conformation but also to expose the most desirable epitopes to elicit a protective immune response against all seven clades of CCHFV (Table 1).

## 6. *Orthobunyaviruses* (Family *Peribunyaviridae*): A Sub-Domain Can Sometimes Be Sufficient to Elicit a Protective Immune Response

The orthobunyaviruses are widespread arthropod-borne viruses commonly associated with central nervous system diseases of increasing importance in humans and animals. La Crosse virus has become the leading cause of pediatric arboviral encephalitis in the USA, responsible for 50–100 cases each year [58] while Oropouche virus is now the second most common arboviral disease, after Dengue fever, in Brazil [59]. Moreover, comparable to most of the other members of the *Bunyavirales* order, orthobunyaviruses are characterized by a tri-segmented single stranded RNA genome. This feature opens the possibility of reassortment, i.e., the exchange of segments between two parental viruses to generate a new virus with potentially altered pathogenic properties (a phenomenon well documented for the appearance of new pandemic influenza virus strains). This possibility is not only theoretical, as the reassortment of the non-pathogenic Bunyamwera and Batai viruses have led to the appearance of the pathogenic Ngari virus, which has been associated with several fatal hemorrhagic fever cases in East Africa [60].

To date, no vaccine against any orthobunyavirus has been tested in clinical trials in humans or animals. Very few studies have investigated the immune response against orthobunyaviruses. In 1995, a DNA-based vaccine against La Crosse virus coding for Gn/Gc glycoproteins was shown to elicit a protective immune response mediated by neutralizing antibodies and CD4+ T cells in a mouse model [61]. More recently, X-ray crystallography studies of Schmallenberg virus, an orthobunyavirus which recently emerged in Europe causing abortion and congenital deformities in newborn ruminants, showed that the spikes protruding at the surface of the virion are formed by an N-terminal extension of the fusion glycoprotein Gc and that this extension is the major target of the neutralizing antibody response [16]. Moreover, immunization of mice with the spike domains efficiently inhibited viremia upon subsequent infection. Given the very similar structural organization of different orthobunyaviruses, the N-terminal variable half of Gc seems to be a promising lead for a subunit vaccine design (Table 1). Following the work done by Nuccitelli et al. on the B Streptococcus pilus [62], one could conceive a recombinant antigen made of the N-terminal variable part of Gc from several orthobunyaviruses fused together. Indeed, Nuccitelli et al. showed that protective antibodies specifically recognize one of the four domains of B streptococcus pilus. They constructed a recombinant protein constituted of the protective domain of each one of the six major variants of the pilus and showed that this chimeric protein protected mice against subsequent infection challenge.

## 7. *Arenaviruses* (Family *Arenaviridae*): Differences in the Target-Cell Entry Pathway Result in Different Neutralization Mechanisms within the Same Family

Arenaviruses are zoonotic viruses infecting a wide range of animals from fish and reptiles to mammals. Since 1933, eight arenaviruses have been identified that cause mild or severe human illness. They are classified according to their geographic distribution and phylogeny into the Old World (such as Lassa, lymphocytic choriomeningitis and Lujo viruses) and the New World (such as Machupo and Junin viruses) arenaviruses [63]. Human infection occurs through direct or indirect contact with infected rodents (such as ingestion of contaminated food or inhalation of contaminated aerosolized particles), but secondary person-to-person transmission has also been described with Lassa, Machupo, and Lujo viruses [64]. Both Old World and New World viruses cause significant disease burden in their endemic areas of circulation. Symptoms range from flu-like illness to meningoencephalitis and hemorrhagic shock. It is estimated that Lassa virus may be responsible for about 100,000 to 300,000 cases and 5000 to 6000 deaths each year, primarily in Sierra Leone, Liberia, Guinea, and Nigeria. In South America, New World Arenaviruses occur less frequently but their mortality reaches 15 to 35% [65]. The only approved vaccine is a live attenuated vaccine, named Candid#1, that prevents infection from Junin virus, one of the most prevalent New World arenaviruses in South America [66]. However, in spite of sequence similarities between New World arenaviruses, this vaccine does not protect against infection from other New World viruses.

**Figure 4:**
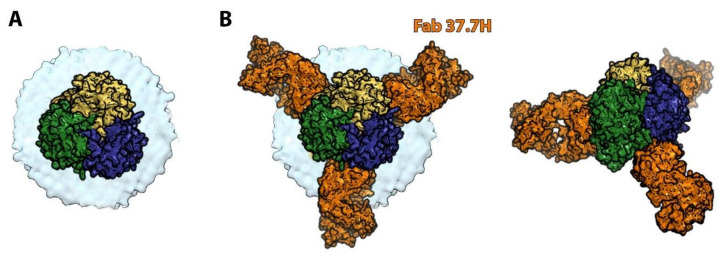
The Lassa virus glycoprotein spike alone and in complex with a neutralizing mAb. (**A**) Representation of Lassa virus glycoprotein trimer. Each monomer is composed of GP1 and GP2 and is displayed in a different color (green, yellow and blue). The membrane is drawn in light blue. (**B**) Modelling of the interaction of Lassa virus spike with a neutralizing antibody displayed in orange. mAb 37.7 binds two GP monomers near the base of the GP trimer.

Arenaviruses are quite different from the other bunyavirus families mentioned in this review. Their genome is composed of two ambisense RNA segments which encode four proteins: the matrix protein, the RNA-dependent RNA polymerase complex, the viral nucleoprotein and a glycoprotein complex (GPC) that is later cleaved into three sub-units named GP1, GP2 and SSP, a transmembrane stable signal peptide essential for viral infectivity [67]. The GPC remains non-covalently associated, forming a trimer of GP1/GP2/SSP heterotrimers (Figure 4A). GP2 is the fusion protein (belonging to the class I fusion proteins) whereas GP1 binds receptor and determines the tropism of the virus. Arenavirus GPC organization displays several differences with the previously discussed bunyavirus glycoproteins. Three of the most important ones for vaccine design are: (1) Arenavirus GPCs form isolated spikes at the surface of the virion; (2) as in the other class I fusion proteins, GP2 requires a proteolytic cleavage event to adopt its metastable fusion-competent state; (3) Arenavirus GPCs are extensively glycosylated, which shield the virion and impair the neutralizing antibody response efficiency [68].

As the sole antigen on the viral surface, the Arenavirus GPC is the primary target of the protective humoral immune response and the focus for vaccine design efforts. It is interesting to note that the target and mode of action of neutralizing mAbs seems to differ between Old World and New World arenaviruses. Indeed, all described New World arenavirus-neutralizing mAbs target GP1 and, more precisely, the receptor binding site of GP1 [69]. Most of them neutralize the virus by mimicking receptor binding. It is important to note that, as the receptor binding site varies significantly among the New World arenaviruses, these antibodies lack the usual breadth associated with this mode of neutralization. Instead, most of the Lassa virus-neutralizing human mAbs bind quaternary epitopes only displayed on prefusion GPC trimers and formed by both GP1 and GP2 subunits [70]. Co-crystallization experiments of Lassa virus GPC ectodomain with a neutralizing mAb showed that the antibody anchors two GPC monomers together within a trimer, thus blocking the conformational changes required for entry (Figure 4B) [71]. These differences in epitope recognition and neutralization mechanism may be, in part, linked to differences in the multistep entry pathway of Old World arenaviruses (involving a receptor switch in the endosome and possible extensive conformation changes of GP1) which have not been described in New World arenaviruses [67].

Of note, GP2-specific mAbs have been isolated after infection/immunization with either Old World or New World arenaviruses: in line with the sequence conservation of GP2 these antibodies are able to bind several species of arenaviruses. However they are not neutralizing.

Taken together these data indicate that single subunit-based vaccines (such as the GP1 subunit alone) may be successful against New World but not Old World arenaviruses. An Old world arenavirus vaccine candidate should contain an engineered immunogen displaying a stabilized prefusion conformation of the trimeric GPC. Furthermore, as shown by Kathryn M. Hastie and colleagues, site-selective deglycosylation of vaccine proteins can improve their immunogenicity [69].

## 8. Conclusions

With the development of human B cell technologies to identify neutralizing antibodies and of structural biology combining high resolution data of X-ray crystallography with electron microscopy, rational vaccine design is now possible: antigens can be stabilized in the most beneficial conformation and modified to favor the presentation of the most relevant antigenic epitopes. Although limited by inherent constraints (notably the difficulty of co-crystallizing a viral antigen with a neutralizing antibody) and the fact that the analyzed structures are inherently static, which do not allow them to take into account the in vivo dynamic behavior of surface proteins (therefore an epitope identified on a crystal structure may not translate to an effective vaccine in vivo), this structure-based antigen design strategy is of particular value to build rapid responses to emerging new threats, as evidenced with the coronavirus disease 2019 pandemic. As climate change and irreversible environmental damage worsens globally, spillover from arbo- and rodent-borne viruses, such as bunyaviruses, will become an increasing concern. Structural vaccinology applied to bunyaviruses is an essential tool for preparedness to cope with future outbreaks. Indeed, if a pan-bunyavirus vaccine does not seem feasible, the design of a vaccine for each of the four families discussed above is now a realistic objective. As the target of the protective humoral response against each family has been identified, the next challenge will be to characterize the common epitopes within different strains/clades to design broadly protecting vaccines. Since bunyaviruses have complicated ecological cycles involving a wide range of vectors and animal reservoirs, spreading across large geographic areas, human outbreaks are difficult to predict. Combination of knowledge gained with structural vaccinology tools and use of a vaccine platform allowing rapid development and large-scale deployment (such as DNA/RNA vaccine platforms) will therefore be essential to limit future outbreaks.

## Figures and Tables

**Table 1:** Further challenges in structural vaccinology for the design of vaccines against bunyaviruses. NWA: New World Arenaviruses; OWA: Old World Arenaviruses; GPC: glycoprotein complex.

Bunyavirus Genus	Target of Neutralizing/Protecting mAbs	Further Challenges
*Orthohantavirus*	Gn/Gc spike	Design of a soluble “frozen” spike to prevent “breathing”
*Phlebovirus*	Gn	Further epitope mapping on different phleboviruses’ Gn to identify potential conserved target of neutralizing mAbs
*Nairovirus*	Gc and GP38	Solving of Gn/Gc spike structure and further mapping of the synergic epitopes in all seven clades Solving the structure of Gc in complex with synergistic mAbs or bsAbs Elucidation of GP38′s role in protection
*Orthobunyavirus*	Gc N-terminal half (spike domains)	Further epitope mapping on Gc spike domains to identify potential conserved targets of neutralizing mAbs
*Arenavirus*	GP1 for NWA Trimeric GPC for OWA	NWA: deciphering the molecular basis of increased virus neutralization potency and breadth on GP1 OWA: engineering a stable prefusion trimeric GPC with site-selective deglycosylations
